# Clinical and economic comparative analysis of laparotomy versus laparoscopy in the first gastric bypass surgeries in a bariatric and metabolic surgery service in a city in southern Brazil

**DOI:** 10.1590/0100-6991e-20233513-en

**Published:** 2023-07-19

**Authors:** RICARDO REICHENBACH, AUGUSTO SGARIONI, MARIA CAROLINA GULLO, HENRIQUE JOÃO GIOVANARDI, GISELE SILVA MOURA

**Affiliations:** 1 - Hospital Geral de Caxias do Sul, Cirurgia Digestiva - Caxias do Sul - RS - Brasil; 2 - Pontifícia Universidade Católica, Pós-Graduaçao - Porto Alegre - RS - Brasil; 3 - Universidade de Caxias do Sul, Medicina - Caxias do Sul - RS - Brasil; 4 - Universidade de Caxias do Sul, Economia - Caxias do Sul - RS - Brasil

**Keywords:** Bariatric Surgery, Laparotomy, Laparoscopy, Economics, Costs and Cost Analysis, Cirurgia Bariátrica, Laparotomia, Laparoscopia, Economia, Custos e Análise de Custo

## Abstract

**Introduction:**

this paper aims to evaluate the main direct and indirect costs of the first laparotomies and laparoscopies in bariatric surgeries with a clinical-economical retrospective and cross-sectional analysis from 2017 to 2020 at a hospital with specialties besides the basic ones in southern Brazil.

**Methods:**

the study sample included 26 participants. The first 13 laparotomies, and the first 13 laparoscopies performed at the bariatric surgery service of the institution were evaluated. The values evaluated in such comparison analyzed the costs of operation and hospitalization. It is important to highlight that, in addition to the cost benefit, other costs take significance in the health area, such as: cost-utility, cost-effectiveness and cost-minimization, in addition to the cost-opportunity that is reassessed in the observation of the broad context associating all the values raised here. The software used for data analysis was Excel version^®^ 365. The economic analysis was performed evidencing the profile of the patients and the direct and indirect costs involved in each segmentation.

**Results:**

the direct and indirect costs of videolaparoscopy amounted to BRL 10,108.10 and laparoscopy to the amount of BRL 12,568.14*.*

**Conclusion:**

it was concluded that laparoscopy presents more savings in the aspects of all health valuations to the detriment of laparotomy. It was concluded that the videolaparoscopy presents more savings in the aspects of all health valuations than the laparotomy.

## RESUMO

**Introdução:**

o presente estudo tem como objetivo avaliar os principais custos diretos e indiretos das primeiras laparotomias e videolaparoscopias em cirurgias bariátricas em uma análise clínica-econômica, retrospectiva e transversal de 2017 a 2020 em um hospital terciário do sul do Brasil.

**Métodos:**

a amostra do estudo incluiu 26 participantes. Foram avaliadas as primeiras 13 laparotomias e as primeiras 13 videolaparoscopias realizadas no serviço de cirurgia bariátrica da instituição. Os valores avaliados em tal comparação analisaram os custos da operação e da internação. É importante ressaltar que além do custo-benefício, outros custos tomam significância na área da saúde. São eles: o custo-utilidade, o custo-efetividade e o custo-minimização, além do custo-oportunidade que é reavaliado na observação do contexto amplo associando todas as valorações aqui levantadas. O software utilizado para a análise dos dados foi o Excel^®^ versão 365. A análise econômica foi realizada evidenciando o perfil dos pacientes e os custos direto e indireto envolvidos em cada segmentação.

**Resultados:**

os custos diretos e indiretos da videolaparoscopia somaram o montante de R$ 10.108.10 e da laparoscopia o montante de R$ 12.568,14.

**Conclusão:**

concluiu-se que a videolaparoscopia apresenta mais economia nas vertentes de todas as valorizações em saúde em detrimento da laparotomia.

## INTRODUCTION

Obesity has a multifactorial cause and a growing incidence in Brazil and worldwide. According to the Brazilian Ministry of Health, in 2019^1^, 52% of the Brazilian population was overweight and 18% was already considered obese. Its treatment involves nutritional and psychological managements, physical exercise, and drug therapies. However, a portion of patients undergoing such interventions do not respond to these therapeutic maneuvers, and may require a more effective intervention, the procedure known as bariatric surgery^2^. Of the various possible surgical techniques, there are two ways to perform them, by laparotomy and by laparoscopy.

Laparotomy is an older technique, with an incision in the abdominal wall between 15 and 20 centimeters to create a visual field for surgeons. Laparoscopy, on the other hand, consists of a more modern technique in which five smaller incisions are made, between five and 12 millimeters each, allowing a visual field with a camera and the entry of carbon dioxide and instruments to perform the procedure.

The resources of the Brazilian Public Unified Health System (SUS) are scarce, and their use requires optimization of inputs and technical procedures. According to Ordinance N^o^. 5 of 2017^3^, laparoscopic bariatric surgery was incorporated as a procedure that could be performed via SUS. The particularity that justifies this study lies in the value of fund transfers to hospitals for surgical materials, which remain the same as for laparotomy. Laparoscopy, however, requires more expensive materials, such as staplers and special hemostatic forceps. Within this reality, studies that seek to compare the total expenses of each surgical technique and that encompass all the complexity of the costs must be carried out to justify changes in the amounts and fractions of the transfer for each surgical step, which includes materials, hospitalization costs, drugs, and time of temporary incapacity to labor, for example.

In Brazil, there are no studies comparing the listed techniques. In the databases that index scientific articles, there are only two main studies with this assessment by the year 2021^4^ ^,^ ^5^. It is necessary, however, to evaluate the performance of such techniques in the Brazilian reality. The present study therefore aims to evaluate, as a primary outcome, the main direct and indirect costs of the first bariatric laparotomies and laparoscopies in a retrospective analysis of the years 2017 to 2020, in a tertiary hospital in Southern Brazil. As a secondary outcome, the objective is to analyze the socioeconomic profile of the patients in the sample.

## METHODS

This is a cross-sectional, retrospective study, with a clinical-economic analysis of the prevalence of the first laparotomy and laparoscopy surgeries performed at the Bariatric and Metabolic Surgery Service of the General Hospital of Caxias do Sul - RS (HG). This study was approved by the Ethics in Research Committee, registered under protocol 39222020500005341/2021. The patients belonging to this study were those who underwent the first bariatric Roux-en-Y gastric bypasses by laparotomy in the first half of 2017, and by laparoscopy, in the first half of 2020, at the same service. We excluded patients who did not fit this profile from the sample. We extracted the data from the Database prepared with the Billing Sector with the direct hospital costs of each patient at the HG.

The same surgical team performed both laparoscopic and laparotomic procedures. Authors A and H conducted the surgeries. The database was structured by the researchers A and G. After completing the database, the researchers M and R performed the statistical analysis. There is no conflict of interest on the part of the authors. The study sample included 26 participants.

Since the laparoscopic surgeries added up to 13 until the elaboration of this research, we performed a comparative analysis with the first 13 laparotomies. The values evaluated in this comparison analyzed the costs of the operation and hospitalization. The feasibility of laparoscopic surgeries was possible through a partnership between the staple supplier company and HG, the company providing such staples for approximately 30% of their market value for the feasibility of developing studies involving costs.

In this way, the costs that the HG had on these laparoscopic surgeries were accounted for and that does not reflect the costs of the procedure that other hospitals would have. For this reason, the costs obtained were compared by adding the cost value without the discount to offer an approximate of the real value, without discount. We also evaluated the economic impact of days away from work activities of patients as indirect costs. For this calculation of indirect costs, were evaluated days away from work activities based on the data reported on the form for each patient. In laparotomy, this period varies from 60 to 90 days; in laparoscopy, from 16 to 21 days.

According to Brazilian law, in the first 15 days of leave, the worker’s remuneration is paid by the employer, this being the indirect cost of the private sector and not varying from one technique to another, since in both the leave is longer than 15 days. As of the sixteenth day, this cost becomes the responsibility of the public sector, via the National Social Security Institute (INSS), an autarchy of the Brazilian government that receives contributions for the maintenance of the General Social Security Regime. The difference is in the time of payment of the benefit during the days of leave, which ranges from up to six days in laparoscopy, and up to 75 days in laparotomy.

Work leave is paid according to the benefit salary, which corresponds to 91% of the average of all contributions made by the insured person in accordance with the Allowance for Temporary Disability, under Law 8213/916. The salary of each patient was self-reported.

Values in Brazilian currency (Reais - BRL) were transformed into minimum wages in effect in the year of surgery, so the update is directly linked to increases in the minimum wage in force in 2021 (BRL 1,100.00 per month). 

The calculated values of the surgeries followed the SUS table, which was not updated during the present study. The values, therefore, remained current, which justified the non-correction for inflation.

The included data were evaluated and then imported into the Stat/Transfer software, where they were divided according to segmentation of laparotomy and laparoscopy, sub-segmentation of the patients’ profile, hospital costs, and costs of sick leave.

### Statistical analysis

We used Excel®, version 365. The economic analysis included the profile of the patients, as well as the direct and indirect costs involved in each segmentation. The direct costs calculated were the cost of the procedure performed, medications used, and days of hospitalization, whilst the indirect ones were the days away from work activities that each surgical technique requires, borne by the INSS.

## RESULTS

There were no deaths in either group. The evaluation of the primary outcomes revealed that laparoscopy had a mean hospital stay of 2.5 days, and laparotomy, 3.8 days. The direct and indirect costs of each technique can be seen in Table 1.

**Table 1:** Direct and indirect costs.

Technique	Laparoscopy	Laparotomy
Direct costs	BRL 6,720.77 Physicians 29.75% Surgery and materials 52.2% Anesthesia 9.05% Infirmary 8.98%	BRL 6,269.14 Physicians 31.9% Surgery and materials 43.75% Anesthesia 9.69% Infirmary 14.64%
Staplers market cost	BRL 4,233.33	BRL 1,016.00
Indirect costs	BRL 424.00	BRL 6,299.00
Total	BRL 7,144.77	BRL 12,568.14
Correction to market cost	BRL 10,108.10	BRL 12,568.14

Source: authors.

The unintentional postoperative surgical outcomes that were not included in the cost calculation of each procedure revealed that in the laparoscopy sample, no patient evolved with postoperative complications. In the laparotomy one, however, three patients had complications, two with incisional hernia and one with stenosis of the gastroenteroanastomosis. Table 1 presents the direct and indirect costs of the bariatric procedures.

Complication costs were not calculated, since the sample was too small for this type of analysis.

## DISCUSSION

The results of the study demonstrate that laparoscopy was associated with a shorter hospital stay, faster recovery, no complications, higher market value corrected costs, but lower total costs when compared with laparotomy, due to the days of transitory illness indemnification. The difference in the profile of the two samples proved to be statistically insignificant.

Laparoscopy requires an average of five staples, and laparotomy, four. The laparoscopic specific staples are the most expensive part of the procedure. The partnership established between the company supplying the staples and HG made it possible for the staples in each surgery to fall from BRL 4,233.33 to BRL 1,270.00 per procedure, approaching the cost of conventional staplers used in laparotomy, that is, approximately 30% of the value of laparoscopy ones.

Hospital costs with correction to market values reveal that laparoscopy is more expensive for hospitals, but in the sum of total costs, including absence from work activities, this technique is shown to be more economical. Cost-effectiveness, according to the Ministry of Health^7^: 

“[...] are studies in which costs and benefits are calculated in monetary values, making it possible to determine whether a new technology or health intervention generates a net benefit to society. Because the value of all consequences is expressed in monetary values, these evaluations allow the comparison not only of health programs and interventions that produce different consequences, but also of health programs with other interventions outside of health.” (p. 42)

Considering the general economic outcomes in each technique used, both in the case of the partnership signed between the HG and the supplier company, and in the case of the usual market values, the cost-effectiveness proved to be favorable to laparoscopy.

In addition to the cost benefit, other costs are relevant to health area. These are the cost-utility, cost-effectiveness, cost-minimization, and the opportunity cost that is reassessed in the observation of the broad context, associating all the valuations raised here.

The cost-utility specifically observes the related quality of life, using quality of life measurement units, such as shorter postoperative recovery time for each technique.

The cost-utility is favorable to the laparoscopy technique. Although the results of the techniques are similar regarding clinical outcome, patients undergoing laparoscopy have a faster postoperative recovery, shorter hospital stay, and lower risk of postoperative complications.

Cost-effectiveness is used when interventions have similar clinical outcomes, differing not only in terms of costs, but also in terms of expected effects. This type of analysis measures the cost in monetary units divided by a non-monetary or natural unit, such as “years of life saved”.

These intervention-oriented economic evaluations compare two strategies that, in this work, would compare treating the patient in a surgical or conservative approach. Patients undergoing bariatric surgery have necessarily already gone through an attempt at remission of the disease through a conservative approach.

Bariatric surgery patients necessarily have favorable cost-effectiveness for surgery if they do not die from the procedure or until they remain in disease remission. Cost-minimization, on its turn, calculates the difference in costs between alternative interventions that are assumed to produce equivalent results. It is maximized when the patient is triaged for one or another bariatric surgery technique. The availability of bariatric surgery allows the best technique to be indicated for each patient’s clinical profile. Having this option allow for the best cost-minimization.

Finally, the opportunity cost is an aspect of cost analysis within the concept of scarcity. According to Mankiw^8^, “The opportunity cost of an item is what one gives up when getting it”, that is, when one decides, one needs to be aware of the opportunity cost that accompanies that decision, which will be foregone to the detriment of this decision. This analysis takes shape as there is a growing shortage in the potential to offer a quality health service as the SUS remuneration table is not updated as quickly as inflation. This context requires that public spending be seen in its entirety.

Thus, when deciding on laparoscopic surgery, laparotomy will be dispensed with, a less costly surgery at the hospital level, but in return for this waiver, more economical results will be obtained considering public expenses, in addition to saving with complications that are more present in laparotomy and better quality of life for the population undergoing bariatric surgery.

The studies by Nguyen et al.^4^ and Paxton and Matthews^5^ present the outcomes of laparoscopy and laparotomy in bariatric surgeries in North American health centers and corroborate the results of the present study, demonstrating that laparoscopy had lower hospital costs. The small number of studies that assess the cost-effectiveness of laparoscopy and laparotomy can be explained by most studies present in relevant indexes paying more attention to the cost-effectiveness assessment of robotic surgery techniques to the detriment of laparoscopy in bariatric surgery.

Indirect costs were calculated using the maximum number of sick days given to patients in each group, and the base salary was based on the patient’s own report, which could present biases in the calculated values. As the object of this study was not to present a reliable value of the cost of sick leave due to its small sample and because it is a study that justifies prospective research with a large sample, this bias was disregarded.

We did not evaluate the economic profile of complications. The complication that most commonly generates additional costs for a new surgical intervention at a bariatric surgery service is incisional hernia. In the laparotomy group, two patients developed postoperative incisional hernia and had to undergo correction surgery. In the laparoscopy group, no patient developed postoperative incisional hernia. In the total sample of laparotomies performed at the Bariatric Surgery Service of Hospital Geral de Caxias do Sul from 2017 to 2021, approximately 162 patients underwent laparotomy and, of these, 17 (10%) developed postoperative incisional hernia, corroborating studies on the performance of laparotomy versus laparoscopy. Such numbers are close to the ones by Nguyen et al.^4^, who present postoperative incisional hernia as an important complication of the laparotomy technique.

Paxton and Matthews^5^ also point out that patients undergoing laparotomy are more prone to incisional hernia, in addition to a higher prevalence of fistulas, wound infection, and extra-intestinal effects, such as deep venous thrombosis, pulmonary embolism, pneumonia, and intra-abdominal abscess.

The general complications of laparotomy are higher than those of laparoscopy in the evaluated sample (Table 2). However, it is worth noting that, as these are the first surgeries of each surgical method, surgeons could be on a learning curve regarding the techniques with the structures available at the Bariatric Surgery Service of the Hospital Geral de Caxias do Sul, which could easily be a confounding bias, and even justified the non-evaluation of the economic impact of complications’ corrections.

**Table 2:** Complications in the evaluated sample.

Complication	Laparoscopy	laparotomy
Incisional hernia	0	2
Stenosis	0	1
Fistula	0	0

Source: authors.

With technological advances, the laparoscopic approach in bariatric surgery increasingly demonstrates a safer and less costly alternative to society. However, for this technique to be widely performed via SUS by the various bariatric surgery services in Brazil, we need studies with large samples, which may legitimize a reorganization of fund transfers for each stage of the surgery, making the technique available in a more comprehensive and democratic way.

The laparoscopic results for bariatric surgery in this study show an economic advantage in the inclusion of this surgical technique broadly by SUS. Although the materials are more expensive in laparoscopy than in laparotomy, the general costs of the laparotomy technique overlap with laparoscopy, increasing the predictive value of the need to reorganize fund transfers at each stage of the procedure.

## CONCLUSION

The present study demonstrates that bariatric surgery using the laparoscopy technique is more economical than laparotomy in terms of health valuations.

Although the study is retrospective and has a small sample, it serves to increase the predictive value on the subject and justifies a future prospective analysis with a broader sample. Prospective studies with large samples can, therefore, provide enough data for changes in surgical methods and procedures to be democratically made possible for the entire SUS user population, with the reorganization of fund transfers. There was no conflict of interest on the part of the authors.
